# Development of prognostic model incorporating a ferroptosis/cuproptosis-related signature and mutational landscape analysis in muscle-invasive bladder cancer

**DOI:** 10.1186/s12885-024-12741-5

**Published:** 2024-08-06

**Authors:** Sida Hao, Zitong Yang, Gang Wang, Guofeng Cai, Yong Qin

**Affiliations:** 1 Department of Urology, Zhejiang Integrated Traditional Chinese and Western Medicine Hospital, Hangzhou, 310003Zhejiang China; 2 Department of Urology, The Fourth Affiliated Hospital, Zhejiang University School of Medicine, Yiwu, Zhejiang China; 3 Department of Urology, Affiliated Hangzhou First People’s Hospital, Xihu University School of Medicine, Hangzhou, Zhejiang China

**Keywords:** Muscle-invasive bladder cancer, Ferroptosis, Cuproptosis, TCGA, Nomogram, Prognostic model

## Abstract

**Background:**

Muscle-invasive bladder cancer (MIBC) is a prevalent and aggressive malignancy. Ferroptosis and cuproptosis are recently discovered forms of programmed cell death (PCD) that have attracted much attention. However, their interactions and impacts on MIBC overall survival (OS) and treatment outcomes remain unclear.

**Methods:**

Data from the TCGA-BLCA project (as the training set), cBioPortal database, and GEO datasets (GSE13507 and GSE32894, as the test sets) were utilized to identify hub ferroptosis/cuproptosis-related genes (FRGs and CRGs) and develop a prognostic signature. Differential expression analysis (DEA) was conducted, followed by univariate and multivariate Cox’s regression analyses and multiple machine learning (ML) techniques to select genetic features. The performance of the ferroptosis/cuproptosis-related signature was evaluated using Kaplan–Meier (K–M) survival analysis and receiver-operating characteristics (ROC) curves. Mutational and tumour immune microenvironment landscapes were also explored. Real-time quantitative reverse transcription polymerase chain reaction (RT-qPCR) experiments confirmed the expression patterns of the hub genes, and functional assays assessed the effects of SCD knockdown on cell viability, proliferation, and migration.

**Results:**

DEA revealed dysregulated FRGs and CRGs in the TCGA MIBC cohort. SCD, DDR2, and MT1A were identified as hub genes. A prognostic signature based on the sum of the weighted expression of these genes demonstrated strong predictive efficacy in the training and test sets. Nomogram incorporating this signature accurately predicted 1-, 3-, and 5-year survival probabilities in the TCGA cohort and GSE13507 dataset. Copy number variation (CNV) and tumour immune microenvironment analysis revealed that high risk score level groups were associated with immunosuppression and lower tumour purity. The associations of risk scores with immunotherapy and chemical drugs were also explored, indicating their potential for guiding treatment for MIBC patients. The dysregulated expression patterns of three hub genes were validated by RT-qPCR experiments.

**Conclusions:**

Targeting hub FRGs and CRGs could be a promising therapeutic approach for MIBC. Our prognostic model offers a new framework for MIBC subtyping and can inform personalized therapeutic strategies.

**Supplementary Information:**

The online version contains supplementary material available at 10.1186/s12885-024-12741-5.

## Introduction

Bladder cancer stands as the most common malignancy within the urinary system [1]. In the U.S., it ranks as the sixth most prevalent cancer, with 83,190 new cases annually, and is the ninth leading cause of cancer-related deaths [2]. Men are notably more affected by this disease than women [1, 2]. Muscle-invasive bladder cancer (MIBC) is an aggressive form of urothelial carcinoma (UC) characterized by tumour invasion into the detrusor muscle [3]. It exhibits significant heterogeneity and prognostic variability, posing considerable challenges to current therapeutic approaches [4]. Despite the application of multiple treatment modalities including surgical resection, chemotherapy, and radiotherapy, the 5-year survival rate for MIBC remains at only 40–60% [5]. Therefore, there is an urgent need to develop a validated prognostic model and discover novel therapeutic targets.

Recent advancements in programmed cell death (PCD), particularly ferroptosis and cuproptosis, have greatly enhanced our understanding of cancer progression [6–8]. Ferroptosis is an iron-dependent cell death marked by lipid peroxidation [9, 10], while cuproptosis involves copper-induced protein aggregation and cell death [11, 12]. The crosstalk between these two cell death pathways suggests that targeting both types of cell death could enhance the effectiveness of cancer treatments [13]. A study on hepatocellular carcinoma identified a signature combining ferroptosis-related or cuproptosis-related genes (FRGs, CRGs) (G6PD, NRAS, RRM2, SQSTM1, SRXN1, TXNRD1, and ZFP69B) for prognosis and therapy prediction [14]. In breast cancer, another study established a prognostic model based on five CRGs and FRGs (ANKRD52, HOXC10, KNOP1, SGPP1, and TRIM45). These genes are significantly associated with immune infiltration and can predict patient outcomes [15]. However, the combined effect of ferroptosis and cuproptosis on MIBC survival and treatment outcomes remains unclear.

Given the heterogeneity of MIBC and the urgent need for effective treatment options, immunotherapy, including immune checkpoint inhibitors (ICIs), has gathered widespread attention as a novel anti-tumour strategy. The expression of programmed death-ligand 1 (PD-L1) in UC patients is a significant predictive biomarker for improved overall survival (OS) with immune checkpoint inhibitors (ICIs) compared to chemotherapy, particularly in PD-L1–positive patients [16, 17].

Multi-omics approaches, encompassing genomics, transcriptomics, proteomics, and metabolomics, have been proven to be pivotal in advancing cancer research [18]. By analysing transcriptomics and genomics data, researchers identified a ferroptosis/cuproptosis-related signature, which was used to develop a prognostic model for patients with MIBC. Integrating multi-omics analysis techniques, this study investigates the roles of ferroptosis and cuproptosis in MIBC progression. Furthermore, it evaluates the signature’s predictive value for immunotherapy efficacy and drug activity, thereby improving disease management.

## Methods

The workflow of this study is shown in Additional file 1: Figure S1.

### Patient cohorts

Transcriptional gene expression matrix (RNA-sequencing [RNA-seq] data) and clinical information of patients in The Cancer Genome Atlas (TCGA)-BLCA project were obtained from the Genomic Data Commons (GDC) Data Portal (https://portal.gdc.cancer.gov/). The segmental CNV data of the TCGA-BLCA cohort were simultaneously downloaded from the cBioPortal for Cancer Genomics software platform (https://www.cbioportal.org/) [19]. Two probe intensity datasets, GSE13507 [20] and GSE32894 [21], were accessed through the Gene Expression Omnibus (GEO) repository (https://www.ncbi.nlm.nih.gov/geo/). Detailed data sources, sample inclusion–exclusion criteria, and data processing methods are provided in Additional file 2. The final TCGA cohort (training cohort 1) included a total of 384 tumour samples (segmental CNV profiles: 380 tumour samples) and 17 histologically normal adjacent tumour (NAT) tissues. GSE13507 dataset comprised 165 primary bladder cancer patients, and GSE32894 dataset consisted of 224 UC cases.

### Cell culture

Human bladder cancer cell lines T24 and UM-UC-3, along with the normal human uroepithelial cell line SV-HUC-1, were obtained from the American Type Culture Collection (ATCC, Manassas, VA, USA). These cell lines were cultured in media supplemented with 10% fetal bovine serum (FBS) and 1% penicillin/streptomycin, under a 5% CO_2_ atmosphere at 37 °C. The specific media used were Roswell Park Memorial Institute medium (RPMI)-1640 for T24 cells, Minimum Essential Medium (MEM) for UM-UC-3 cells, and F12K for SV-HUC-1 cells. Each cell line was tested using three samples, and all experiments were carried out in triplicate.

### RT-qPCR experiment

Total RNA from T24, UM-UC-3, and SV-HUC-1 cells was extracted using the FastPure Cell/Tissue Total RNA Isolation Kit V2. Reverse transcription was performed with the HiScript® III 1st Strand cDNA Synthesis Kit (+ gDNA wiper). The real-time quantitative reverse transcription polymerase chain reaction (RT-qPCR) assays utilized the Taq Pro Universal SYBR qPCR Master Mix, purchased from Vazyme (Nanjing, China). GAPDH served as an internal control. Primer sequences for SCD, DDR2, MT1A, and GAPDH are listed in Table 1.

**Table 1:** List of primer sequences for SCD, DDR2, MT1A, and GAPDH (housekeeping gene)

Gene		Sequence	Length (bp)
SCD	Forward	CCCGACGTGGCTTTTTCTTC	20
	Reverse	GCCAGGTTTGTAGTACCTCCTC	22
DDR2	Forward	GTTGGGGAAACGCAGTGGAT	20
	Reverse	GGTCTCCCTTGATGGAGGTTTC	22
MT1A	Forward	CTCTTGCTGTTGCTGATGGG	20
	Reverse	TCGTGAGACCTTCGCTCTTGT	21
GAPDH	Forward	GACAGTCAGCCGCATCTTCT	20
(housekeeping gene)	Reverse	GCGCCCAATACGACCAAATC	20

### Cell Transfection

Three siRNAs targeting SCD and a negative control siRNA (si-NC) were procured from GenePharma (China). Cell transfection was executed using jetPRiME (Polyplus, NY, USA) following the manufacturer’s instructions. Sequences for the siRNAs targeting SCD are provided in Table 2.

**Table 2:** List of primer sequences for three siRNAs targeting SCD and a negative control siRNA (si-NC)

Gene		Sequence	Length (bp)
si-#1	Sense	5’-CUUCGUUUGAAGCAAGAAUTT-3’	21
	Antisense	5’-AUUCUUGCUUCAAACGAAGTT-3’	21
si-#2	Sense	5’-CAAGUCCUCUACCGAAUGATT-3’	21
	Antisense	5’-UCAUUCGGUAGAGGACUUGT-3’	20
si-#3	Sense	5’-GAUGAGCAGUCCAAAGCAUTT-3’	21
	Antisense	5’-AUGCUUUGGACUGCUCAUCT-3’	20
si-NC	Sense	5’-UUCUUCGAACGUGUCACGUTT-3’	21
	Antisense	5’-ACGUGACACGUUCGGAGAATT-3’	21

*NC* Negative control

### CCK-8 Assay

Cells were seeded in 96-well plates at a density of 1000 cells per well. CCK-8 solution (10 μL) at a 1:10 dilution with Dulbecco’s modified eagle medium (DMEM) (100 μL) was added to each well for measuring cell viability and proliferation rate. Cell proliferation was assessed at 0, 24, 48, and 72 h post-seeding using a microplate reader to measure optical density at 450 nm.

### Transwell assay

Cell invasion assays were conducted using 24-well Transwell chambers (Corning, NY, USA). Approximately 40,000 cells, suspended in serum-free medium, were placed into the upper chamber, while the lower chamber was filled with medium containing 20% FBS as a chemoattractant. Migrated cells were fixed with polyformaldehyde, stained with crystal violet, and photographed under a fluorescent microscope.

### Wound healing assay

Cells were seeded in 12-well plates at a density of 3 × 10^5^ cells per well and grown to confluence. A scratch was made in the monolayer using a tip, and cells were then washed with PBS to remove detached cells. Cells were cultured in complete medium. The representative of the scratch, from the same field, was photographed at 0 and 24 h post-scratch.

### Statistical analysis

Statistical analyses were conducted using R (v. 4.3.2, R Foundation for Statistical Computing, Vienna, Austria). All *p*-values were derived from two-sided tests. Detailed descriptions of the statistical methodologies are available in Additional file 2.

### Comparison of baseline characteristics

Bivariate associations between OS status and patients’ baseline characteristics in the three datasets were tested. Pearson’s chi-squared test was used for differential analysis of categorical variables, and Yates’ continuity correction was applied if at least one expected cell count was less than 5 but greater than or equal to 1 (for 2 × 2 contingency table only). Fisher’s exact test was used when at least one expected cell count was less than 1.

### Identification of DEGs and co-expressed genes

The FRGs and CRGs are detailed in Additional file 3: Tables S1 and S2. After running voom on TCGA count matrix, differentially expressed genes (DEGs) between 17 MIBC biopsies and 17 NAT tissues were detected using the “limma” package. DEGs were defined based on an absolute log_2_(fold-change) [log_2_(FC)] greater than 2 and a false discovery rate (FDR)-adjusted *p*-value below 0.001.

Co-expression relationships were assessed using Spearman’s rank correlation coefficient from the “Hmisc” package [22]. This analysis was performed on a log_2_-transformed matrix of tumour samples, with significance defined as a *p*-value below 0.05 and an absolute correlation coefficient (|r|) above 0.4.

### Establishment of ferroptosis/cuproptosis-related signature

To screen for hub genetic features, univariate and multivariate Cox’s proportional hazards (PH) regression and machine learning (ML) techniques were employed to analyse the normalized TPM expression matrix through a rigorous selection process. The ML methods included the least absolute shrinkage and selection operator (LASSO)-penalized Cox’s model, support vector machine-recursive feature elimination (SVM-RFE), and bidirectional stepwise regression. Details are shown in Additional file 2.

Expression data of hub genes were used to perform a multivariate Cox’s regression analysis to compute an individualized genetic risk score for each patient in the training cohort and test set. The risk score was calculated using the following equation:

1 $$Risk {score}_{i} = \sum_{j-1}^{n}{\beta }_{ij} \times {expr}_{\beta ij}$$

where *risk score*_*i*_ represents the risk score for sample *i*, *j* represents the number of hub gene, *β*_*ij*_ represents the multivariate Cox’s regression coefficient value of each hub gene, and *expr*_*ij*_ stands for the normalized TPM value of each gene in sample *i*.

Correlations between the expression levels of hub BMGs and the risk scores were examined using the “Hmisc” package [22]. Patients were then categorized into low and high risk score level groups based on the median value. Expression of hub genes between the two groups was compared using independent *t*-test. This method was also applied to external validation datasets (GSE13507 and GSE32894), adjusting the cutoff points due to differences in sequencing platform.

### Development and validation of nomogram-based prognostic model

Univariate Cox’s regression analysis was utilized to assess the associations between the ferroptosis/cuproptosis-related signature and patients’ demographic and clinical characteristics. Significant variables were then selected for further evaluation using bidirectional stepwise regression from the “stats” package [23]. TNM stage was excluded after that to prevent multicollinearity. These significant predictors, alongside the ferroptosis/cuproptosis-related signature, informed the development of a nomogram-based prognostic model for predicting OS.

The associations between the expression level of each hub gene and the risk score and independent demographic and clinical predictors were examined. Two-sample independent *t*-test was employed for variables with normal distribution, while Wilcoxon’s rank-sum test was utilized for variables that did not follow a normal distribution. The performance of the nomogram-based predictive model was assessed using various metrics, including C-index for discrimination, integrated Brier score (IBS) for prediction accuracy, and calibration curves for model calibration. Additionally, decision curve analysis (DCA) [24] was used to evaluate utility of models.

### Construction of biological interaction networks

To seek potential interactions between DEGs, a protein–protein interaction (PPI) network was constructed using the STRING (Search Tool for the Retrieval of Interacting Genes/Proteins) database (v. 12.0, https://string-db.org/) [25]. In the network, the nodes stand for the proteins and the edges represent the interactions. The minimum interaction score was set to a medium confidence level of 0.400 to ensure the reliability of the interactions. Additionally, the STRING database was used to perform overrepresentation analysis (ORA) using KEGG pathway gene sets (herein referred to as “ORA–KEGG”). Significant pathways associated with cancer (FDR-adjusted *p* < 0.05) were visually distinguished in the PPI network by assigning distinct colours to the corresponding nodes for clear annotation. In addition to the PPI networks, resultant links between pairs of co-expressed DEFRGs and DECRGs from correlation analysis were integrated to generate a co-expression network.

### Functional enrichment analysis

To evaluate the enrichment of DEGs among functional terms, ORA was conducted using Gene Ontology (GO) annotation (hereinafter referred to as “ORA–GO”). The Reduce and VIsualize Gene Ontology (REVIGO) web tool (http://revigo.irb.hr/) was used to remove any redundancy of top 30 significantly enriched GO terms [26]. Based on semantic similarities, after merging and replacing the representative subset, non-redundant GO terms were displayed in a semantic space with a cutoff value *C* of 0.7.

Moreover, gene set enrichment analysis (GSEA) was carried out on Hallmark gene sets (hereinafter referred to as “GSEA–Hallmark”) and KEGG gene sets (hereinafter referred to as “GSEA–KEGG”), with genes ranked either by log_2_(FC) or Spearman’s r for single-gene GSEA. The top 20 enriched Hallmark terms, ordered by their |enrichment scores|, were visualized as a facet dot plot using the “ggplot2” package [27]. The KEGG pathway database comprises a set of pathway maps drawn by hand. For human (*Homo sapiens*) species, these maps are divided into six distinct types: metabolism, genetic information processing, environmental information processing, cellular processes, organismal systems, and human diseases [28]. KEGG pathways categorized under the human diseases category were excluded from this analysis. Subsequently, the “aPEAR” package [23] was used to leverage similarities between the significantly enriched KEGG pathways. Afterwards, the “ggraph” package [29] and “ggforce” package [30] was utilized to represent the result as a network of interconnected clusters. Enrichment analysis was conducted utilizing the “clusterProfiler” package [31]. A functional term or pathway was deemed statistically significant with an FDR-adjusted *p*-value less than 0.25 and *p*-value less than 0.05.

### GSVA

The gene set variation analysis (GSVA) algorithm provided by the “GSVA” package [32] was used to measure the activity of the top 20 significant gene sets from GSEA–Hallmark (hereinafter referred to as GSVA–Hallmark). Additionally, the immune infiltration score (IIS) was determined based on 24 immune cell types, which included various subsets of macrophages, dendritic cells (DCs), B cells, cytotoxic cells, eosinophils, mast cells (MCs), neutrophils, nature killer (NK) cells, and T cells. This score, derived from the research of Senbabaoglu et al. [33]., aimed to estimate the abundance and infiltration of these immune cells within the tumour environment. The GSVA scores for these 24 immune cells were calculated using the “GSVAutils” package [34]. Furthermore, the antigen processing and presenting machinery (APM) score was calculated from mRNA expression levels of APM genes to evaluate the efficiency of antigen processing and presentation, a critical factor influencing ICI response. Finally, the tumour immunogenicity score (TIGS) [34] was computed by integrating the APM score with the tumour mutational burden (TMB), providing a comprehensive assessment of the tumour’s potential responsiveness to immunotherapy.

### Exploration of gene mutation pattern

Analysing MAF (MAF) files is crucial in cancer genomics to encapsulate and summarize mutation data across various samples. Oncoplots were created to visualize the somatic mutation landscape of the top 20 mutated genes within cohorts stratified by risk score level using the “ggplot2” package [27]. Tumour mutation burden (TMB) was calculated by summing up all missense, insertion/deletion, and frameshift variants within each tumour sample using the following equation [34]:

2 $$TMB=1n \left(\frac{whole exome mutation}{38} +1\right)$$

CNVs are pivotal in the oncogenesis and progression of malignant tumours [35], necessitating detailed analyses of gene-level and segmental CNVs across 384 samples. To depict the genomic landscapes of CNVs and their associations with OS, a lollipop was visualized via the “ggplot2” package [27] and a circos diagram using the circos tool (v. 0.69–9; tools version: 0.23) [36]. Moreover, the GISTIC (Genomic Identification of Significant Targets in Cancer) 2.0 algorithm [37], provided by the GenePattern tool (https://cloud.genepattern.org/) [38], was applied to pinpoint significant genomic regions underpinning cancer, highlighting amplified or deleted segments through GISTIC scores and *q*-values. These focal regions were further intersected with the Gencode GTF file (gencode.v43.annotation.gtf.gz) using Bedtools (v. 2.27.1) [39] to align genomic data with gene annotations effectively. Gene mapping to position on the chromosome was performed using the genome reference consortium human build 38 (GRCh38/hg38).

### Analysis of tumour microenvironment

Cells in tumour microenvironment consist of tumour cells, stromal cells and immune cells [40]. The Estimation of STromal and Immune cells in MAlignant Tumours using Expression data (ESTIMATE) method was applied using the “tidyestimate” package [41] to analyse these components in 384 MIBC samples from the TCGA database. The ESTIMATE method provided scores reflecting immune and stromal cell infiltration, overall ESTIMATE scores, and tumour purity estimates. Additionally, the Cell-type Identification By Estimating Relative Subsets Of RNA Transcripts (CIBERSORT) algorithm was used to analyse the gene expression matrix constructed from TPM data, employing the CIBERSORT R script (v. 1.04, Stanford University, CA, USA) [42] with 1000 permutations, offering a detailed profile of immune cell composition within the TME.

These scores and immune cell profiles were statistically analysed between two risk score groups using Wilcoxon’s rank sum test via the “rstatix” package [43]. The association between expression levels of hub genes and immune cell fractions differing in two risk score level groups was assessed using Spearman’s rank correlation analysis with the help of “Hmisc” package [22]. All statistical analyses were performed with a significance threshold set at a p-value of less than 0.05 to ensure robustness of the findings.

### Prediction of immunotherapy efficacy and drug activity

Tumours that respond well to ICIs are referred to as immunologically “hot” tumours, while those that do not respond favourably are termed immunologically “cold” tumours. The Tumour Immune Dysfunction and Exclusion (TIDE) score for each sample in the TCGA cohort was calculated using the TIDE database (http://tide.dfci.harvard.edu/) [44]. TIDE and TIGS scores were compared between two risk groups using Wilcoxon’s rank sum test provided by the “rstatix” package [43]. Correlations between the risk score, immune infiltration score (IIS), and 17 immune checkpoints (detailed in Additional file 4: Table S3) were examined using the “correlation” package [45], with a significance level set at a *p*-value below 0.05.

Additionally, drug activity prediction was performed based on the data from the RNAactDrug database (http://bio-bigdata.hrbmu.edu.cn/RNAactDrug), an extensive resource for exploring drug activity and RNA expression level correlations, integrating data from GDSC, CellMiner, and CCLE databases [46]. The analysis focused on the association between RNA molecules and drug activity, selecting records with an FDR-adjusted *p*-value below 0.25 and a *p*-value less than 0.05. Compounds showing at least two significant correlations with hub genes were visualized as bubble plots using the “ggplot2” package [27].

### Differential analysis of RT-qPCR data

Gene expression was presented using a modification of the 2^−ΔΔCt^ method. To analyse the differential relative expression of the hub genes and si-SCD in cancer and normal cell lines, *p*-values from pairwise *t*-test for independent groups were calculated and then adjusted using the FDR method by the “rstatix” package [43]. Ct values in samples for each mRNA were visualized as a bar plot using the “ggplot2” package [27]. An FDR-adjusted *p*-value less than 0.05 was considered significant for mRNA, and less than 0.0001 for siRNA.

### Analysis of repeated-measures data

Linear mixed-effects model (LMM) provided by the “mmrm” [47] was introduced to fit linear mixed-effects models. Then, “emmeans” [48] package was utilized to assess the differential impact on cell viability (%) exerted by three siRNAs targeting SCD in cancer cell lines against that of si-NC. An FDR-adjusted *p*-value less than 0.0001 at the time point of 72 indicated a statically significant effect of the SCD-targeting siRNAs on cell viability compared to the control.

## Results

### Baseline characteristics of participants

The TCGA cohort consisted of 384 participants diagnosed with MIBC, including 102 females (26.56%) and 282 males (73.44%). The median age of the participants was 68.00 [IQR: 60.00, 76.00] y/o. Of these, 213 participants (55.47%) were alive at the time of analysis, while 171 (44.53%) had succumbed to the disease. The analysis of survival rates revealed no statistically significant difference between female and male participants (*p* > 0.05). **Additional file 5: Tables S4–6** provide a comprehensive summary of the demographic and pathological details for MIBC patients in the TCGA cohort (training set), as well as for the GSE13507 dataset (test set 1) and the GSE32894 dataset (test set 2).

### Identification of DEFRGs and DECRGs between tumour samples and NATs

**Fig. 1:**
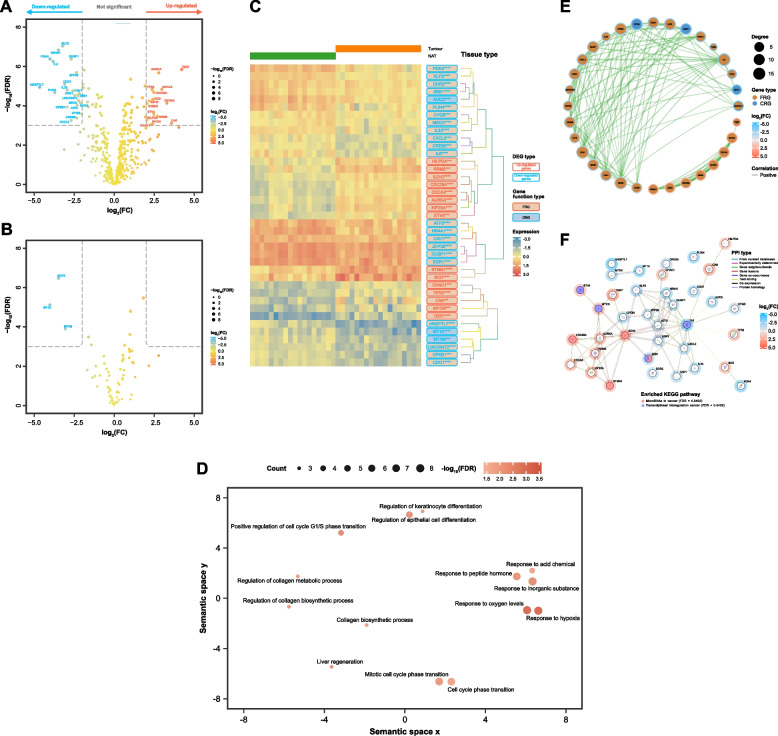
Identification of differentially expressed ferroptosis-related (DEFRGs) and cuproptosis-related genes (DECRGs). **A** Volcano plot depicting DEFRGs. **B** Volcano plot showing DEFRGs. DEGs (down-regulated and up-regulated genes) were determined in 17 MIBC specimens vs. 17 paired normal adjacent tumour (NAT) tissues using a threshold of |log_2_(FC)|> 2 and a false discovery rate (FDR)-adjusted *p*-value < 0.001. **C** Heatmap illustrating expression pattern of 36 DEFRGs and 3 DECRGs across 17 MIBC specimens and 17 paired NAT tissues. The rows of the heatmap represent genes, and the columns represent samples. Each cell is colourized based on the level of expression of that DEFRG or DECRG in that sample. **D** Bubble plot showing the GO cluster representatives in a two-dimensional space. Each circle indicates a representative cluster. The colour of the circles represents -log_10_(FDR) value of the GO analysis. The size of each circle indicates the count of genes involved in the GO term. **E** Co-expression network of DEFRGs and DECRGs (blue: down-regulated, red: up-regulated, size: degree; sky blue: negative, tomato red: positive correlation). **F** PPI network composed of 37 nodes (proteins) and 75 edges (interactions) from STRING (confidence: 0.400, *p* < 0.0001; sky blue halo: down-regulated, tomato red halo: up-regulated).*** *p* < 0.001, **** *p* < 0.0001

Differential expression analysis (DEA) between 17 MIBC specimen and 17 paired NAT tissues from the TCGA cohort revealed 833 down-regulated and 470 up-regulated genes (*p* < 0.001 and |log_2_(FC)|> 2). Out of these, 21 DEFRGs were up-regulated and 15 were down-regulated (Fig. 1A and Additional file 6: Table S7). Three DECRGs exhibited down-regulation (Fig. 1B and Additional file 6: Table S8). In Figs. 1A and 1B, volcano plots depict the results of DEA for FRGs and CRGs respectively. In Fig. 1C, the heatmap colours show the expression of each DEG across 34 samples.

REVIGO was applied to summarize long lists of GO terms from DEGs in the training cohort. Relevant biological processes identified include regulation of keratinocyte differentiation, response to hypoxia, regulation of collagen metabolic process, and positive regulation of cell cycle G1/S phase transition (Fig. 1D).

Spearman’s correlation analysis (Fig. 1E and Additional file 7: Table S9) explored the interconnections among these genes, revealing a co-expression network of 32 genes, including 29 differentially expressed FRG (DEFRGs) and 3 differentially expressed CRG (DECRGs). All correlation pairs exhibited positive correlations, with genes such as ZEB1 (DECRG, 17 partners) and IL6 (DEFRG, 15 partners) demonstrating higher connectivity.

The PPIs were analysed for the known and predicted interactions between DEGs. The PPI network (Fig. 1F and Additional file 8: Table S10) comprised 37 nodes (proteins) and 75 edges (interactions). ATF3 (FRG) demonstrates the highest number of significant correlations with 10 partners. Notably, the connection between AURKA (DEFRG, 9 partners) and MYCN (DEFRG, 6 partners) had an exceptionally high combined score, indicating a potentially crucial role in cellular ferroptosis. Besides, the interaction between MT1A (DECRG, 1 partner) and MT1M (DECRG, 1 partner) may reflect a co-evolved function in cuproptosis. PPI analysis also revealed significant enriched KEGG pathways, including the microRNAs in cancer and transcriptional misregulation cancer.

### Selection of hub genes

Univariate Cox’s regression analysis was performed on DECRGs and DEFRGs in the training set. Genes with statistical significance (*p* < 0.05) and a C-index greater than 0.5 were identified as DECRGs and DEFRGs associated with OS. Ten genes, including AOC3, MEG3, EGR1, SCD, CAV1, CDO1, CREB5, DDR2, MT1A, and ZEB1, were found to have significant impacts on OS (Additional file 9: Table S11).

**Fig. 2:**
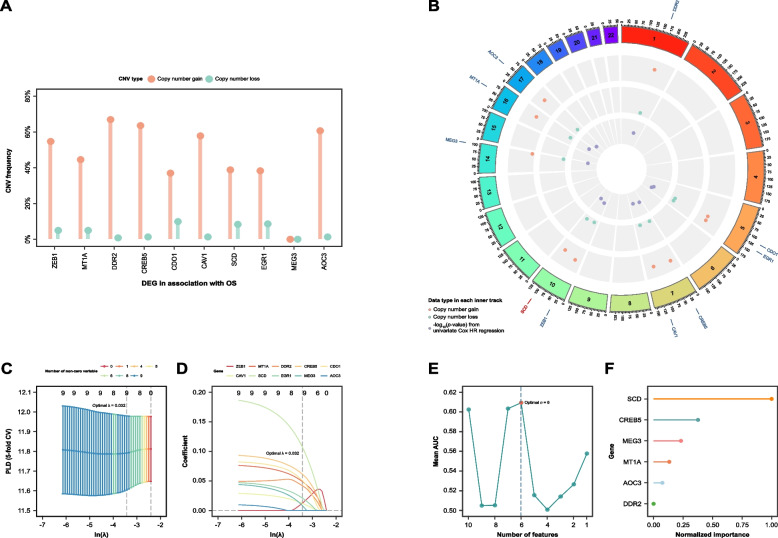
Selection of hub genes. **A** Lollipop chart illustrating CNV frequencies of DEFRGs and DECRGs in association with overall survival (OS). Bar length shows copy number gain/loss percentage. **B** Circos plot demonstrating chromosomal CNV distribution of 21 DEFRGs and DECRGs in association with OS. Tracks from outer to inner: genomic locations, percentage of copy number gain, percentage of copy number loss, -log_10_(*p*-value) from univariate Cox’s analysis. **C** Plot indicating the best penalty parameter (optimal λ = 0.032) selection by cross-validation (CV) partial likelihood deviance (PLD) of the least absolute shrinkage and selection operator (LASSO)-penalized Cox’s regression. **D** Plots showing LASSO-penalized Cox’s regression coefficients over different values of ln(λ). **E** Mean discriminatory performance of feature subsets of each size selected by linear support vector machine recursive feature elimination (SVM-RFE) regression model. CV mean area under the curve (AUC) was plotted against subset size. The red dot represents that the optimal number of selected variables is six. **F** Lollipop plot showing the normalized importance value of each of the six selected features in the linear SVM model. *y/o* Years old

Chromosomal distribution and CNV frequencies of the ten DECRGs and DEFRGs associated with OS were analysed (Figs. 2A and 2B). Notably, DDR2, located on chromosome 1, had the highest copy number gain at 66.93% and the lowest loss at 0.78%. CREB5, located on chromosome 7, also demonstrated a substantial gain of 63.54% with a loss of 1.30%. Interestingly, MEG3, located on chromosome 14, showed no copy number variations, indicating stability in its genomic copy number.

Two ML methods with fivefold cross-validation were used for gene selection, enhancing robustness and preventing overfitting. LASSO-penalized Cox’s regression identified nine genes with non-zero coefficients at the optimal penalty parameter (λ = 0.032) (Figs. 2C and 2D). The SVM-RFE algorithm further refined the selection to six genes based on their maximal mean AUC value (Figs. 2E and 2F). The genetic features identified by both ML methods were subsequently considered as candidates in a multivariate Cox’s PH regression analysis. Bidirectional stepwise selection identified SCD, DDR2, and MT1A as hub genes, which are the most contributive predictors for OS (Additional file 10: Table S12).

### Establishment of ferroptosis/cuproptosis-related prognostic signature

**Fig. 3:**
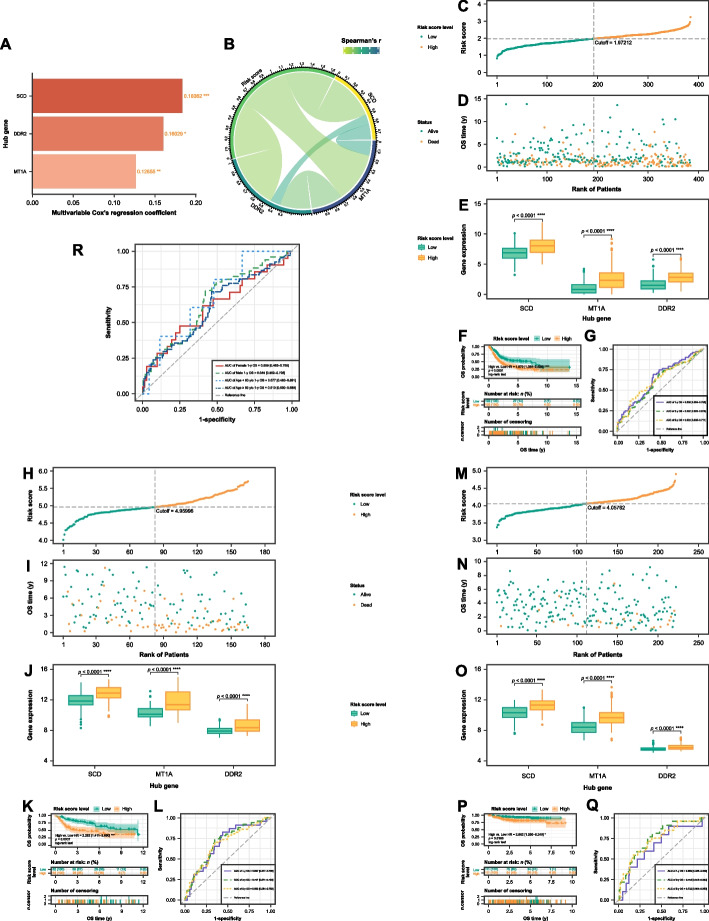
Establishment of the ferroptosis/cuproptosis-related signature and validation of its prognostic value. **A** Bar plot showing multivariate Cox’s regression coefficient for each hub gene. **B** Chord diagram of Spearman’s correlation between expression level of hub genes and risk score. The distribution of each patient’s risk score ordered from low to high in (**C**) TCGA cohort, (**H**) GSE13507 dataset, and (**M**) GSE32894 dataset. Patients in each dataset were divided into two risk score level groups based on each dataset’s median. Scatter diagram of overall survival (OS) time against patients’ rank of risk score in (**D**) TCGA cohort, (**I**) GSE13507 dataset, and (N) GSE32894 dataset. Boxplot depicting the expression level of each hub gene was significantly different between the two risk score level groups in (**E**) TCGA cohort, (**J**) GSE13507 dataset, and (**O**) GSE32894 dataset. The independent sample *t*-test was applied for comparing the differences, and the *p*-values were FDR-adjusted. Low risk score level tissues marked in blue colour and high risk score level samples marked in red. Kaplan–Meier (K–M) plot demonstrating elevation in OS probability in (F) TCGA cohort, (K) GSE13507 dataset, and (**P**) GSE32894 dataset. Plots of the time-dependent receiver-operating characteristics (ROC) curves for the risk score prognostic model for 1-, 3-, and 5-year OS and the corresponding area under the curve (AUC) values in (**G**) TCGA cohort, (**L**) GSE13507 dataset, and (**Q**) GSE32894 dataset. (**R**) Plots of the time-dependent ROC curves for the risk score prognostic model in each age and gender subgroups of the TCGA cohort for 1-year and the corresponding AUC values. Each AUC value is represented in the legend as the estimated value [95% CI]. * *p* < 0.05, *** *p* < 0.001, **** *p* < 0.0001

The development of a ferroptosis/cuproptosis-related prognostic signature was confirmed through a multivariate Cox’s PH model using data from the TCGA cohort. The risk score calculation was based on a linear weighted combination of hub gene expression levels: *Risk score* = (0.18362 × *SCD expr*) + (0.16029 × *DDR2 expr*) + (0.12655 × *MT1A expr*). The multivariate Cox’s regression coefficients for each hub gene were displayed using a forest plot in Fig. 3A. The links between risk score and gene expression were visualized through circos charts in Fig. 3B. All of three hub genes had a strong correlation with the risk score.

### Performance evaluation of the prognostic signature

The performance evaluation of the prognostic signature was conducted across multiple datasets, including TCGA and two GEO datasets (GSE13507 and GSE32894). The distribution of risk scores is presented in an ascending order, aligned with patient rankings in the training set (Fig. 3C) and the two test sets (Figs. 3H, and 3M). Risk score cutoff was set based on the median risk score of each cohort, classifying samples into low and high risk score level groups. Samples with a risk score below the threshold were categorized into the low risk score level group, and those above into the high risk level group. The model’s discrimination capability between different OS outcomes was effectively showcased through scatter diagrams, which plotted the OS-time against patients’ risk score rankings. This demonstrated strong discrimination within the TCGA cohort, as shown in (Fig. 3D), and was further validated in the datasets represented in (Figs. 3I and 3N). Significant differences in the expression levels of the three hub genes between high and low risk score level groups were consistently observed across all datasets, as shown in Figs. 3E, 3J, and 3O.

K–M survival analysis confirmed the effectiveness of the risk score level in predicting survival across all datasets (Figs. 3F, 3K, and 3P). The performance of the univariate model incorporating risk score performance was further assessed using time-dependent ROC curves (Figs. 3G, 3L, and 3Q), demonstrating strong predictive efficacy. Additionally, this signature also exhibited good discriminatory ability across demographic subgroups of MIBC patients, as illustrated in Fig. 3R.

### Development and validation of nomogram-based model

**Fig. 4:**
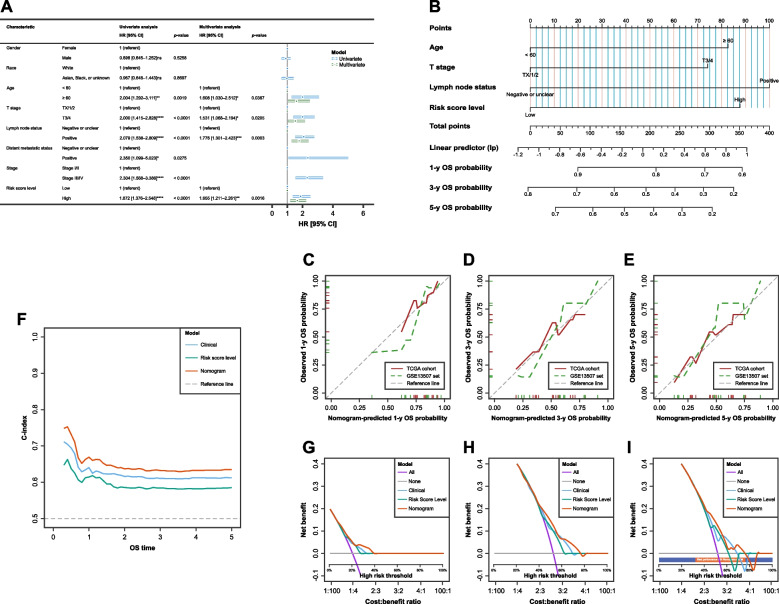
Development, and internal and external validation of nomogram-based prognostic model incorporating the ferroptosis/cuproptosis-related signature. **A** Forest plot of uni- and multi multivariate Cox’s model for overall survival (OS) based on the TCGA cohort (training set). **B** A nomogram for predicting 1, 3, and 5-year OS possibilities in the MIBC patients relying on the TCGA population. The steps for using the nomogram are (1) determine the individual patient’s point for each predictor (T stage, Lymph node status, and risk score level), (2) draw a straight line upwards from each predictive value to the top point reference line, (3) sum the points from each predictive variable, (4) locate the sum on the total points reference line, and (5) draw a straight line from total points line down to the bottom probability lines to obtain the patient’s likelihood of 1-, 3-, and 5-year OS. The diagram also prints how many linear predictor units there are per point and the number of points per unit change in the linear predictor (lp). Calibration curves of Kaplan–Meier (K–M) vs nomogram predicted (C) 1-, (D) 3-, and (E) 5-year OS for TCGA cohort (red) and GSE13507 dataset (green). (F) Time-dependent c-index curves for the “Clinical”, “Risk Score Level”, and “Nomogram” models. DCA for the “Clinical”, “Risk Score Level”, and “Nomogram” models built to predict (G) 1-, (H) 3-, and (I) 5-year OS probability based on records of patients in TCGA cohort (Orange bar: range where the Nomogram model performs better others, deep blue bar: range not relevant to the Nomogram model). ns not significant, * *p* < 0.05, ** *p* < 0.01, *** *p* < 0.001, **** *p* < 0.0001

In the establishment and validation of a nomogram-based model, univariate and bidirectional stepwise multivariate Cox’s PH regression analyses identified variables significantly associated with OS. The multivariate analyses revealed that older age (≥ 60 y/o vs. < 60 y/o, HR = 1.608 [95% CI: 1.030–2.512], *p* = 0.0367), higher T stage (High vs. Low, HR = 1.531 [95% CI: 1.068–2.194], *p* = 0.0205), lymph node metastasis (Positive vs. Negative, HR = 1.776 [95% CI: 1.301–2.423], *p* = 0.0003), and high risk score level (High vs. Low, HR = 1.655 [95% CI: 1.211–2.261], *p* = 0.016) were significantly associated with worse OS in the TCGA cohort. The results of univariate and multivariate Cox’s PH regression analyses are summarized in Fig. 4A.

A nomogram (Fig. 4B) was developed utilizing these independent prognostic factors to predict 1-, 3-, and 5-year OS probabilities for MIBC patients. This nomogram demonstrated excellent discrimination and calibration in both training and test datasets, with a C-index of 0.660 [95% CI: 0.639–0.681] for the training cohort and an integrated Brier score (IBS) of 0.206 [95% CI: 0.185–0.227]. The performance in the test dataset was consistent, with a C-index of 0.752 [95% CI: 0.724–0.781] and an IBS of 0.166 [95% CI: 0.151–0.181]. The 1000-resampling calibration plots for the training cohort and validation set 1 (GSE13057) at 1, 3, and 5 years demonstrated minimal deviations from the 45° reference line, indicating robust model performance (Figs. 4C–E).

Further internal and external validation showed that the nomogram provided better predictive accuracy than other established Cox’s PH regression models. The time-independent C-index of the nomogram model consistently outperformed other models at any time point, indicating superior discrimination (Fig. 4F). Decision curves confirmed that the nomogram model had the highest net benefit for predicting 1-, 3-, and 5-year OS probability, surpassing all other options (Figs. 4G–I). The orange bar in each decision curve analysis (DCA) plot represents a threshold probability range where the nomogram model outperforms other models, disregarding random noise [24].

The differential analysis performed on the TCGA cohort, investigating the disparities in risk scores and the expression of hub genes across demographic and clinical subgroups, yielded substantial findings (Additional file 11: Tables S13–15). Firstly, gender did not significantly impact the risk score or gene expression levels. In contrast, age demonstrated a marked influence, with patients under 60 showing lower risk score levels and reduced expression of SCD and MT1A genes compared to those aged 60 and above. Higher T stage was associated with elevated risk scores and increased expression of DDR2 and MT1A in advanced T stages. Lastly, the presence of lymph node metastasis correlated with elevated risk scores and DDR2 expression but not with SCD and MT1A.

### Enriched Hallmark Terms and KEGG Pathways

**Fig. 5:**
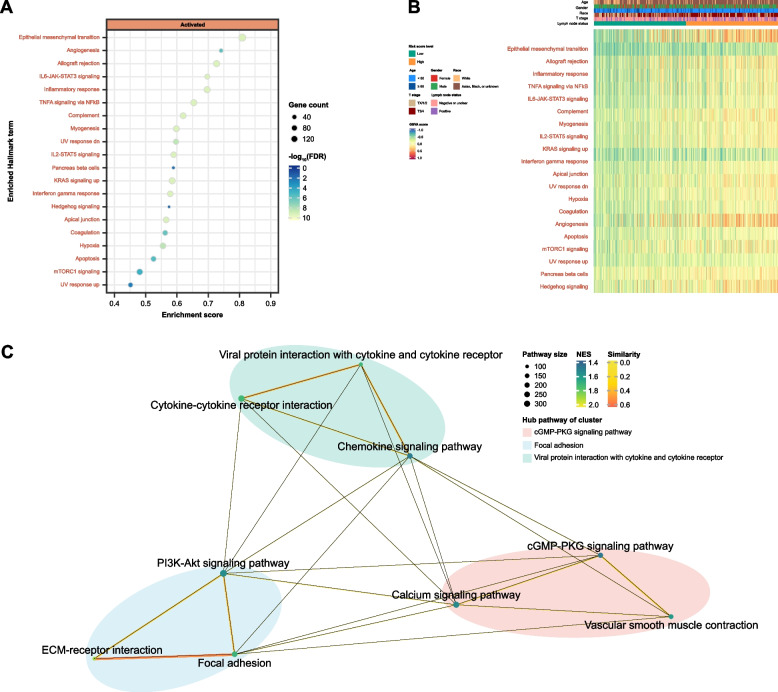
Enriched gene sets associated with risk score level in the gene set enrichment analysis (GSEA) performed on TCGA data. **A** Dot plot depicted the 20 most significantly enriched Hallmark terms ranked by gene ratio. Dot size is proportional to the number of overlapping genes. *p*-values are colour-coded according to the colour scale. **B** Heatmap plots of gene set variation analysis (GSVA) scores across 384 TCGA samples for the 20 most significantly enriched Hallmark gene sets. Red label: activated Hallmark terms. **C** Clustering network of significantly enriched KEGG pathways in the GSEA analysis. The nodes represent the significant KEGG pathways and the edges represent similarity between them and are coloured by normalized enrichment score (NES). The lines connected similar pathways are coloured by similarity

Figure 5A shows the top 20 enriched terms from the GSEA-Hallmark analysis (FDR-adjusted *p* < 0.25 and *p* < 0.05). All of these terms were activated in the high risk score level group. The GSVA method was used to estimate the pathway activity by transforming the input gene-sample expression data matrix into a corresponding gene set-sample expression data matrix (i.e., pathway expression score matrix). Figure 5B is the heatmap illustrating the GSVA scores of the top 20 enriched Hallmark pathways across 384 TCGA samples. Both figures indicate the upregulation of these terms in relation to higher risk of death for MIBC patients. Among them, angiogenesis, IL6-JAK-STAT3, IL2/STAT5, complement system, interferon gamma response, and G2M checkpoint, are crucial for tumour growth, inflammation, and immune system evasion.

The clustering of significant pathways from the GSEA–KEGG in Fig. 5C has identified several groups based on their functional similarities. This analysis highlights the interconnected nature of biological pathways, underscoring how various aspects of cellular functioning are closely related. For instance, pathways involving PI3K-Akt signaling, ECM-receptor interactions, and focal adhesions cluster together, suggesting a shared role in processes such as cell migration, proliferation, and survival. Similarly, the close grouping of the cytokine-cytokine receptor interaction, chemokine signaling pathway, and viral protein interactions with cytokines and their receptors indicates a concerted involvement in immune response and inflammation.

### Somatic mutation and segmental CNV in patients with different risk score levels

**Fig. 6:**
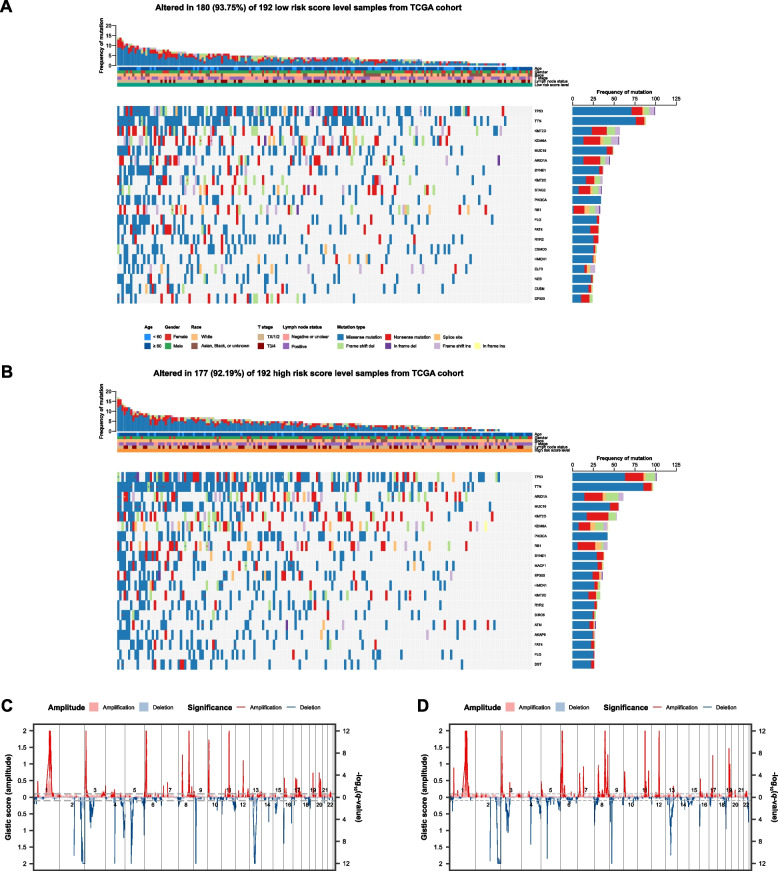
Somatic mutation and copy-number alteration in association with risk score level. **A** MAF mutation oncoplot showing top 20 altered in 180 (93.75%) of 192 low risk score level samples from TCGA cohort. **B** MAF mutation oncoplot showing top 20 altered in 177 (92.19%) of 192 high risk score level samples from TCGA cohort. **C** GISTIC amplification (up, red) and deletion (down, steel blue) plot of 191 TCGA low risk score level samples. **D** GISTIC amplification (up, red) and deletion (down, steel blue) plot of 189 TCGA high risk score level samples

In the low risk score level group, 93.75% samples displayed somatic mutation (Fig. 6A). The most frequently mutated gene was TP53, a well-known tumour suppressor gene, followed by TTN. Other significant genes affected by somatic mutation include KMT2D, KDM6A, and MUC16, which are involved in chromatin modification, histone demethylation, and cell adhesion, respectively. In the high risk score level group, genetic changes were evident in 92.19% of the samples (Fig. 6B). These mutations encompassed missense mutations, nonsense mutations, splice site alterations, as well as frame shift insertions and deletions. The TP53 gene had the highest frequency of mutation, followed by TTN and ARID1A. Other genes that frequently underwent mutations include MUC16, KMT2D, KDM6A, PIK3CA, and RB1.

The data from chromosomal CNVs for both high and low risk score level groups reveal distinct genomic alterations. Participants in the low risk score level group primarily displayed deletions, including genes such as PEX14, RPS6KA1, and ARID1A (Fig. 6C and Additional file 12: Table S16). Conversely, patients in the high risk score level group exhibited a mix of deletions and amplifications across various chromosomal segments, including genes like SLC9A1, HSPA5P1, and MACF1 (Fig. 6D and Additional file 12: Table S17).

### Immune cell landscape and immunotherapy response prediction

**Fig. 7:**
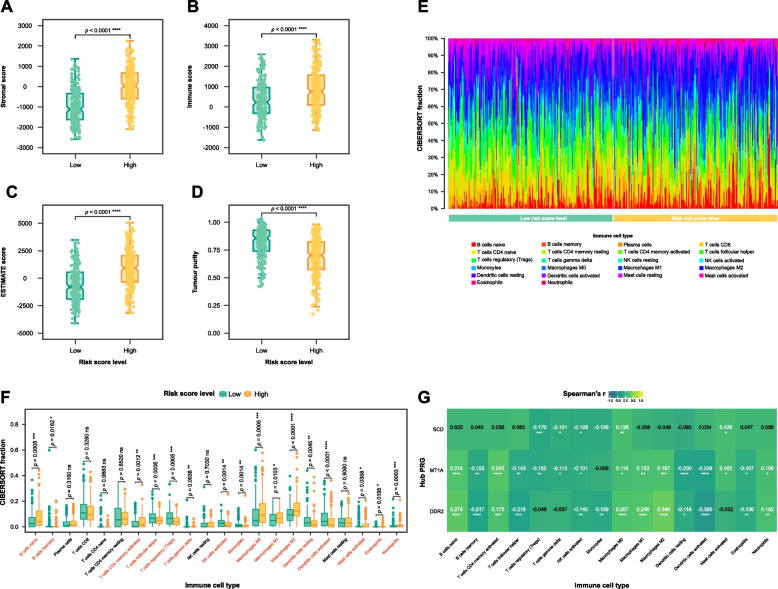
Immune cell landscape. Boxplot reporting the different distributions of (**A**) stromal score, (**B**) immune score, (**C**) Estimation of STromal and Immune cells in MAlignant Tumours using Expression data (ESTIMATE) score, and (**D**) tumour purity between high (*n* = 192, yellow) and low risk score level groups (*n* = 192, green). **E** Bar plot showing composition of 22 infiltrating immune cells in two risk score level subgroups. Fraction values of CIBERSORT immune cells were determined for each patient; each bar represents one patient. **F** Box plot showing the differences in CIBERSORT fractions between tissues from the high risk score level patients (*n* = 192, yellow) and low risk score level participants (*n* = 192, green). **G** Heatmap showing the Spearman’s correlation coefficient between the expression of the three hub genes and immune cell fractions differing in two risk score level groups. ns not significant, * *p* < 0.05, ** *p* < 0.01, *** *p* < 0.001, **** *p* < 0.0001

Differential analysis was conducted on the stromal score (Fig. 7A), immune score (Fig. 7B), ESTIMATE score (Fig. 7C), and tumour purity (Fig. 7D) calculated using the ESTIMATE algorithm. The high risk score level group exhibited significantly increased levels of stromal score, immune score, and ESTIMATE score. Conversely, tumour purity was observed to be lower in this group.

CIBERSORT was also applied to the gene expression matrix in order to infer the relative abundance of 22 tumour-infiltrating immune cells for each sample in the TCGA cohort. Significant differences in the fractions of various immune cells were also observed between the two risk score level groups (Fig. 7E). Notably, the high risk score level group exhibits a significantly lower proportion of B cells memory, T cells follicular helper, T cells regulatory (Tregs), T cells gamma delta, NK cells activated, monocytes, DCs resting, DCs activated, and eosinophils (all *p* < 0.05). Conversely, a higher proportion of B cells naïve, T cells CD4 memory activated, macrophages M0, macrophages M1, macrophages M2, MCs activated, and neutrophils (all *p* < 0.05).

The correlation analysis between gene expression and CIBERSORT immune cell fractions reveals complex interactions within the tumour microenvironment (Fig. 7F). The correlation analysis between gene expression and immune cells reveals significant interactions for hub genes. For SCD, notable correlations include a positive correlation with macrophages M0 and MCs activated (all *p* < 0.05), and negative correlations with monocytes, NK cells activated, T cells gamma delta, and T cells regulatory (Tregs) (all *p* < 0.05). For DDR2, significant positive correlations are seen with B cells naïve, macrophages M0, macrophages M1, macrophages M2, neutrophils, and T cells CD4 memory activated (all *p* < 0.05), while negative correlations include B cells memory, DCs activated, DCs resting, Monocytes, NK cells activated, and T cells follicular helper (all *p* < 0.05). For MT1A, positive correlations are found with B cells naïve, macrophages M0, macrophages M1, Macrophages M2, MCs activated, neutrophils, and T cells CD4 memory activated (all *p* < 0.05), while negative correlations are observed with B cells memory, DCs activated, DCs resting, eosinophils, NK cells activated, T cells follicular helper, T cells gamma delta, and Tregs (all *p* < 0.05).

**Fig. 8:**
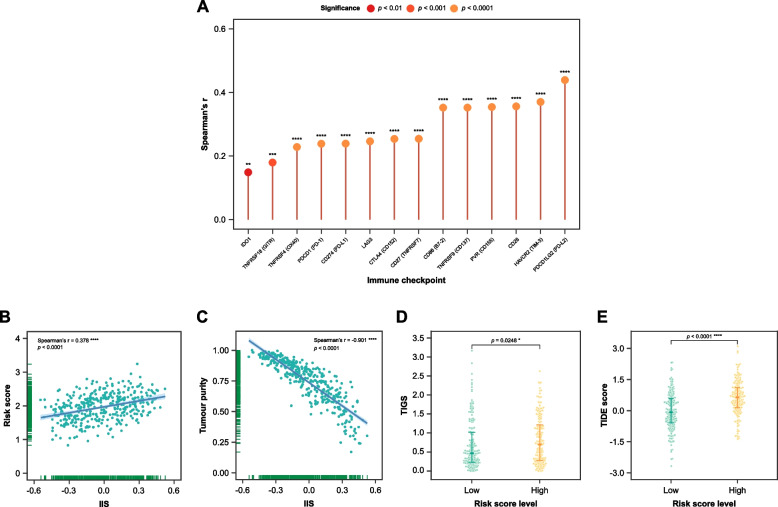
Spearman’s correlation analysis and differential analysis with regards to immune checkpoint inhibitor (ICI) therapy response prediction and tumour immune microenvironment. **A** Lollipop plot showing immune checkpoints that significantly correlate with risk score in TCGA patients. Scatter plot showing (**B**) risk score and (**C**) tumour purity, and immune infiltration score (IIS) correlate among TCGA patients. Beemswarm plot showing significant differences in (**D**) Tumour immunogenicity score (TIGS) and (**E**) Tumour Immune Dysfunction and Exclusion (TIDE) score between the high risk score level (*n* = 192, yellow) and low risk score level groups (*n* = 192, green). * *p* < 0.05, ** *p* < 0.01, *** *p* < 0.001, **** *p* < 0.0001

Additionally, the risk score positively correlated with several immune checkpoint molecules (IDO1, TNFRSF18 [GITR], TNFRSF4 [OX40], PDCD1 [PD-1], CD274 [PD-L1], LAG3, CTLA4 [CD152], CD27 [TNFRSF7], CD86 [B7-2], TNFRSF9 [CD137], PVR [CD155], CD28, HAVCR2 [TIM-3], and PDCD1LG2 [PD-L2]) (Fig. 8A) and IIS (Fig. 8B). The correlation analysis also revealed a positive association between the IIS and tumour purity (Fig. 8C). Meanwhile, the differential analysis revealed that the low risk score level group displayed lower TIDE and TIGS scores (Figs. 8D and E).

### Candidate chemical drugs

**Fig. 9:**
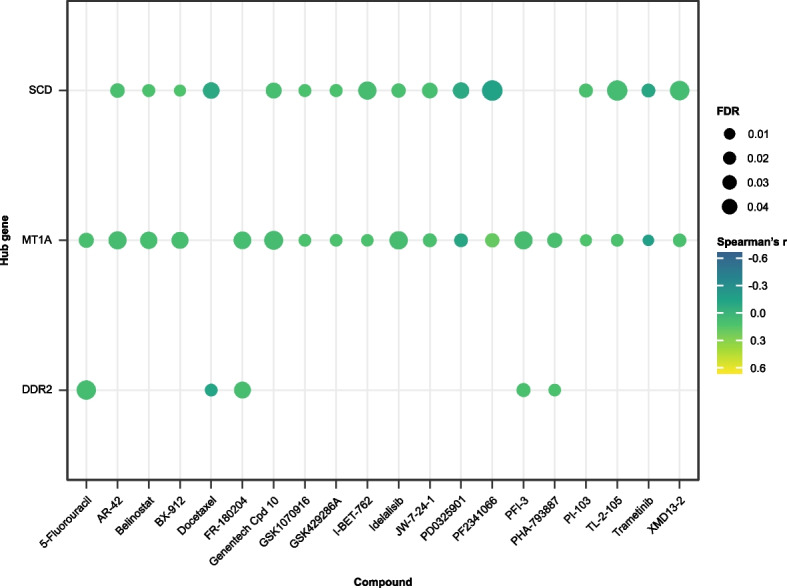
Bubble plot illustrating the correlation between hub genes (y-axis) and compounds (x-axis). Each point’s size is proportional to the false discovery rate (FDR), while the colour gradient indicates the Spearman’s r, representing the correlation between the two variables. The correlation data between drug activity and mRNA expression in cell lines was sourced from RNAactDrug

The study extracted statistically significant correlations between specific chemical compounds and RNA molecules from the RNAactDrug database, which have implications for chemical drug activity (Fig. 9). The data primarily originated from the GDSC database, with an exception for PF2341066, where information was derived from the CCLE. Noteworthy associations were found between the chemotherapy agent 5-fluorouracil and RNA molecules DDR2 and MT1A, suggesting a potential link in drug response. Similarly, the compound AR-42 exhibited significant correlations with MT1A and SCD RNA molecules. The histone deacetylase inhibitor belinostat (PXD101) [49] and PDK1 inhibitor BX-912 also displayed correlations with these RNA molecules. The microtubule inhibitor Docetaxel and the kinase inhibitor FR-180204 were significantly associated with DDR2 and SCD, as well as DDR2 and MT1A, respectively. Innovative compounds, such as Genentech Cpd 10 and GSK1070916, along with the PI3K inhibitor idelalisib, showed significant associations with MT1A and SCD. Notably, PF2341066 demonstrated significant correlations with MT1A and SCD.

### Biological experiments and single-gene GSEA

**Fig. 10:**
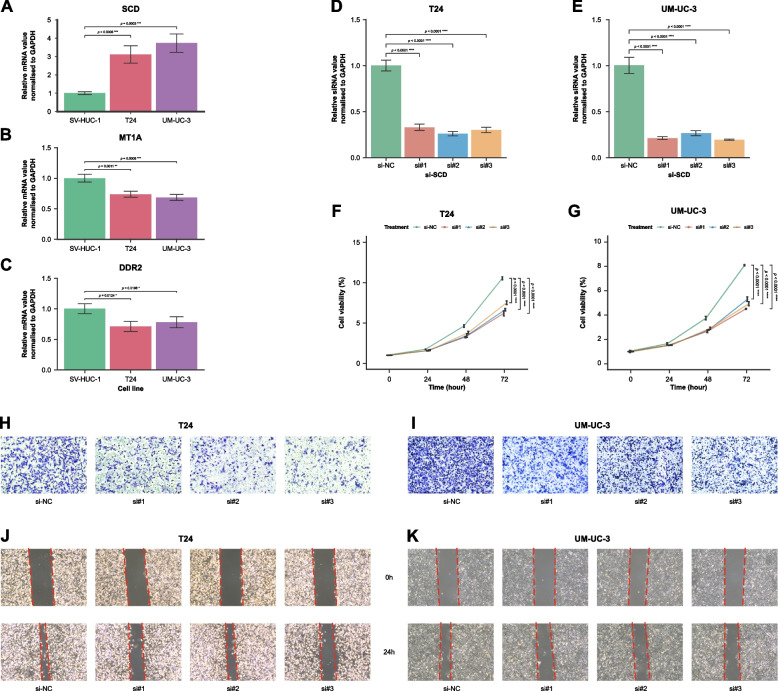
Biological experimental validation and the impact of SCD on cell proliferation, migration, and invasion of human bladder cells. **A**–**C** Bar plot showing the relative expression level of the three hub genes in a normal cell line (SV-HUC-1) and two bladder cancer cell lines (T24 and UM-UC-3). (A) SCD was up-regulated in two cancer cell lines compared to the normal cell line, while (**B**) M1TA and (**C**) DDR2 were down-regulated. The RNA transcription levels of hub genes were evaluated by using the 2.^−ΔΔCt^ method. GAPDH was used as an internal control. Time course of (F) T24 and (**G**) UM-UC-3 cell viability after acute treatment with CCK-8. Morphology of migrating (**H**) T24 and (**I**) UM-UC-3 cells after transfecting siRNA#1, siRNA#2, siRNA#3, and si-NC from the microscopy of the Transwell assay. Fluorescent microscope photos to evaluate wound healing in vitro in the scratch assay using (**J**) T24 and (**K**) UM-UC-3 cells after transfecting siRNA#1, siRNA#2, siRNA#3, and si-NC. Knockdown of SCD may inhibit the proliferation, invasion, and migration of bladder cancer cells. Error bars indicate SD. * *p* < 0.05, ** *p* < 0.01, *** *p* < 0.001, **** *p* < 0.0001

The expression patterns of SCD, DDR2, and MT1A genes in both normal and cancerous human urothelial cells were investigated based on differential analysis of data from the qRT-PCR test. The results revealed that the expression level of SCD was significantly elevated in the T24 and UM-UC-3 cell lines compared to SV-HUC-1 (Fig. 10A). In contrast, the expression levels of DDR2 (Fig. 10B) and MT1A (Fig. 10C) were lower in the T24 and UM-UC-3 cell lines than in SV-HUC-1. These RT-qPCR results align completely with the differential expression patterns observed between MIBC samples and NAT tissues in the TCGA cohort.

In MIBC tissues from the TCGA cohort, single-gene GSEA–Hallmark (Additional file 1: Figure S2a) and GSEA–KEGG (Additional file 1: Figure S2b) analyses for the SCD gene revealed significant activation of multiple pathways, including E2F targets, G2M checkpoint, mTORC1 signaling, MYC targets, unfolded protein response, protein secretion, cholesterol homeostasis, oxidative phosphorylation, PI3K-Akt-mTOR signaling, lipid metabolism, and glycolysis. Notably, both analyses highlighted the enrichment of the mTOR signaling pathway, fatty acid metabolism, protein processing/secretion, and cell cycle regulation. To further explore the role of SCD in UC, RNA interference experiments targeting SCD were performed on the T24 and UM-UC-3 cell lines. The efficiency of gene silencing was verified by RT-qPCR, demonstrating that three distinct siRNA sequences successfully knocked down the expression of SCD. The impact of SCD knockdown on cellular proliferation was assessed using CCK-8 assays. The results indicated a significant reduction in cell proliferation rates in both T24 (Figs. 10D and F) and UM-UC-3 cell lines (Figs. 10E and G) following SCD silencing. Additionally, the effects of SCD knockdown on cell migration abilities were evaluated through Transwell and scratch assays. In both the T24 (Figs. 10H and J) and UM-UC-3 cell lines (Figs. 10I and K), the migratory capacity of cells was significantly impaired after SCD silencing, suggesting a critical role of SCD in the regulation of cell motility in UC.

### Discussion

Through comparative analysis of MIBC samples and paired NAT tissues, 36 DEFRGs and 3 DECRGs were identified. GO enrichment analysis was performed on these DEGs. The results in Fig. 1D display significant key biological processes shared by DEGs, which underline the roles of cell death mechanisms, cell proliferation, differentiation, and tumour microenvironment adaptation in MIBC’s progression.

The interplay between ferroptosis and cuproptosis, two recently identified forms of non-apoptotic cell death, has become an increasingly prominent topic in the field of cancer research [13, 15, 50]. Spearman’s correlation analysis revealed that all three DECRGs were significantly correlated with at least one DEFRG in MIBC. Additionally, DEFRGs and DECRGs were involved in some KEGG pathways associated with cancer.

Disruptions in their metabolic functions can lead to lethal consequences for cancer cells, triggering both ferroptosis and cuproptosis [50]. The mechanism of copper-dependent cell death is closely linked to mitochondrial activity. Cuproptosis often occurs when copper binds to lipoylated proteins in the TCA cycle, leading to protein aggregation, loss of iron-sulfur cluster proteins, and proteotoxic stress, ultimately causing cell death [11, 51, 52]. In addition, a connection between mitochondrial copper accumulation and the initiation of ferroptosis has been established. The intricate interaction mechanisms between ferroptosis and cuproptosis in MIBC indeed call for further investigation through experimental studies [50].

The application of multiple ML techniques and Cox’s regression analysis identified SCD, DDR2, and MT1A as hub genes for MIBC patients. SCD, also known as SCD1, is a key player in lipid metabolism. The regulation of the saturated fatty acids (SFAs) to monounsaturated fatty acids (MUFAs) ratio by SCD1 is not only vital for cellular homeostasis but also implicates its role in cell proliferation and survival [53]. Lipid metabolic reprogramming is considered a hallmark of cancer, and the role of SCD reveals the potential of targeting ferroptosis, a regulated cell death process, as a therapeutic strategy [54, 55]. Experimental evidence shows that targeting SCD with siRNA significantly reduces the proliferation and migration of bladder cancer cells, further confirming its role in cancer progression [56]. DDR2 is critical in bladder cancer progression, affecting proliferation, migration, invasion, metastasis, EMT, and chemotherapy resistance, and its abnormal expression and mutations are linked to aggressive cancer phenotypes, highlighting its potential as a therapeutic target [57, 58]. MT1A, a member of the metallothionein (MT) family, is involved in tumour development, progression, and drug resistance, essential for metal homeostasis and cellular stress protection, with its variable expression across cancer types indicating its potential as a biomarker for cancer diagnosis and prognosis, as well as a target for therapeutic intervention [59].

By utilizing the multivariate Cox’s regression coefficients for the three hub genes and their expression data, a risk score was developed to predict the survival probability of MIBC patients. The results from the two validation sets also confirmed that this variable exhibits good discriminative ability across different datasets. Subsequently, a prognostic model incorporating the ferroptosis/cuproptosis-related signature was developed. The internal and external validation indicated that this model can accurately distinguish between patients with high and low risk score levels. The ability to predict survival probability of the nomogram-based model and its robust performance were also confirmed. As such, the model can be used as an effective tool to assist clinicians in assessing the prognostic risk of MIBC patients.

The differential analysis revealed a significant correlation between elevated SCD expression and more advanced T stages in MIBC patients from the TCGA database. This finding gains further support from the results of single-gene GSEA–KEGG and GSEA–Hallmark analyses, which revealed a significant enrichment of genes associated with SCD expression in fatty acid metabolism pathways. This enrichment implies that SCD upregulation goes beyond a simple correlation, indicating an active role in tumourigenesis by influencing lipid metabolism in a manner conducive to cancer proliferation.

In the TCGA cohort, both single-gene GSEA–Hallmark and GSEA–KEGG analyses suggest that the SCD gene may influence the pathogenesis and progression of MIBC by modulating cell cycle, lipid metabolism, and immune response. This emphasizes the potential of SCD upregulation to not only alter cellular metabolic processes but also engage in signaling cascades that are quintessential for cancer progression. SNORD88C acts as a non-invasive diagnostic biomarker for non-small cell lung cancer (NSCLC), promoting cancer progression by enhancing SCD1 translation through specific RNA modifications, which leads to inhibited autophagy and increased tumour growth and metastasis [60]. Furthermore, another line of research has interpreted the role of FGFR3 in bladder tumours. The increased activity of FGFR3 augments the demand for fatty acid desaturation, a fundamental process for maintaining membrane fluidity and effective cell signaling. This increased requirement is satisfied through the signaling via the PI3K-mTORC1 pathway, resulting in the upregulation of SREBP1 and, consequently, SCD [61].

The pathway enrichment analysis further elaborated on the biological significance of the identified DEGs between the two risk score level groups, revealing their involvement in critical pathways that govern cancer progression and response to therapy. Notably, pathways associated with inflammation and immune response were prominently enriched among these genes, highlighting their integral roles in modulating the cellular mechanisms central to MIBC pathogenesis. The results of enrichment analyses provide a molecular blueprint, offering the potential of targeting these pathways for therapeutic intervention. At the same time, CNV analysis not only highlighted the mutational landscape of these critical genes but also underscored the genetic underpinnings that may contribute to the aggressive nature of MIBC.

Comparing the somatic mutation landscape of the two risk score level groups, TP53 mutations are predominant in both, suggesting its fundamental role in the disease regardless of risk level. The presence of additional mutations in genes such as ARID1A, PIK3CA, and RB1 in the high risk group may provide insights into potential therapeutic targets or biomarkers for stratifying patients based on genetic risk. The GISTIC scores further reveal the potential involvement of these genomic regions in cancer pathogenesis. The segmental deletions in the tumour samples with high risk score level suggest a loss of function in pathways associated with a more aggressive cancer phenotype when intact. The segmental CNVs in the tumour samples with high risk score level may indicate a more aggressive tumour phenotype or reflect the genomic instability characterizing the high risk score level group.

Stratifying patients into high and low risk score level groups based on the ferroptosis/cuproptosis-related signature not only provides a wider perspective of the tumour’s biological behaviours and heterogeneity in mutational landscape, but also guides tailored therapeutic interventions. Tumour purity refers to the proportion of cancer cells within a given tumour tissue sample. Accurate assessment of tumour purity is essential for understanding the respective roles of malignant and non-malignant cells within the tumour microenvironment [62]. Decreased tumour purity in the high risk score level group suggested the samples in this group had a higher proportion of non-tumour cells within their microenvironment. Low tumour purity was also associated with worse prognosis in MIBC [63]. Research has shown low purity tumours may signify chromosomal amplifications or deletions. Moreover, cancer patients with low tumour purity may not benefit from adjuvant chemotherapy [64].

As indicated in the immune infiltration differential analysis, different tumour immune microenvironments might influence tumour progression and patient prognosis. Tumours with high risk score level show decreased proportions of memory B cells, Tregs, T cells gamma delta, activated NK cells, and various DCs, indicating reduced immune surveillance. Meanwhile, these samples also exhibit increased naïve B cells, activated CD4 memory T cells, all macrophage subtypes, activated MCs, and neutrophils, suggesting a pro-tumorigenic environment. Gene-immune cell correlation analysis highlights the roles of SCD, DDR2, and MT1A in modulating immune cell infiltration. SCD correlates with increased macrophages and activated mast cells but decreased monocytes and NK cells. DDR2 and MT1A similarly influence a wide range of immune cells. The three hub genes may promote a pro-tumourigenic and immunosuppressive microenvironment.

The risk score was found to positively correlate with several immune checkpoint molecules, like PD-1, PD-L1, and CTLA-4. This suggests that a higher risk score might be indicative of increased immune checkpoint activity, potentially leading to immune evasion by tumours. While high expression of immune checkpoints often associates with poor prognosis for cancer patients [65, 66], this sign can indicate potential responsiveness to ICI [16, 17]. Moreover, innovative strategies such as the use of reactive oxygen species (ROS)-responsive nanoparticles combined with copper and elesclomol to induce cuproptosis, in conjunction with αPD-L1, have shown enhanced cancer immunotherapy outcomes, further emphasizing the importance of targeting immune evasion mechanisms in cancer treatment [67].

IIS, which quantifies the extent and type of immune cell infiltration within the tumour, was positively correlated with risk score in MIBC. Tumours with higher risk score level not only tended to possess increased expression of immune checkpoints, but also exhibited a more substantial immune cell proportion. This dual characteristic might indicate a complex immune landscape where the immune system is actively engaged yet simultaneously suppressed by the tumour’s immune evasion strategies. The low risk score level group has lower TIGS and TIDE scores compared to another group. Lower TIGS score reflects a lower level of tumour immunogenicity, which has been associated with better survival outcomes in patients not treated with immunotherapy [34]. On the other side, positive TIDE score signifies ICI non-response [34]. It can be concluded that patients in the low risk score level group may have a more favourable immunological profile for responding to ICI therapy.

Through analysis with the RNAactDrug database, some chemotherapeutic drugs were found to be significantly associated with the expression levels of hub genes, offering targeted strategies for the potential treatment of MIBC. Emerging biomarkers and potential therapeutics related to cuproptosis and ferroptosis in cancer provide new enlightenment for treatment. A recent study [68] has demonstrated that inhibiting SCD can trigger both ferroptosis and apoptosis in ovarian cancer cells. This dual mechanism presents a significant barrier to the development of drug resistance, making SCD a promising target for anti-cancer therapy [68]. In addition, since cancer cells have a higher demand for copper compared to normal cells, targeting copper metabolism and cuproptosis-related pathways could selectively kill cancer cells without harming normal cells. Potential therapeutic approaches could involve utilizing copper ion carriers or chelators to regulate cellular copper levels in cells, inducing cuproptosis in cancer cells while sparing healthy cells.

Moreover, the results of differential relative expression of hub genes, SCD, DDR2, and MT1A, in bladder cancer cells compared to normal urothelial cells from RT-qPCR experiments were consistent with those of DEA on transcriptomic data from the TCGA cohort. The experimental validation of the expression patterns of the three hub genes confirms the reproducibility of the molecular alterations associated with bladder cancer.

The precise molecular mechanisms and interactions of these hub genes in MIBC remain a critical knowledge gap. Therefore, it is essential to further elucidate the detailed molecular pathways associated with these hub genes and investigate their potential as biomarkers for early diagnosis and treatment response. Additionally, exploring synergistic treatment combinations that integrate traditional therapies with targeted approaches against FRGs and CRGs could enhance therapeutic efficacy. The advancements in multi-omics technologies and the development of novel targeted drugs hold promise for revolutionizing MIBC treatment, making it more personalized and effective.

While this investigation reveals the significance of FRGs and CRGs in the progression of MIBC, it is not without limitations. The molecular heterogeneity of MIBC poses a challenge in identifying uniform therapeutic targets across different subtypes. Additionally, the utilization of public databases introduces variability in data quality and completeness, potentially impacting study findings. Moreover, this study was limited to experiments at the cell line level. Further research involving human clinical samples is necessary to comprehensively validate the expression pattern of the hub genes. Larger, multicentric studies with diverse MIBC patient cohorts are also needed to confirm the prognostic utility of hub genes. Additionally, exploring the influence of the tumour microenvironment on treatment response in different risk score level groups is crucial. Furthermore, this study did not delve deeply into the roles of hub genes in mediating cuproptosis and ferroptosis in MIBC. Future research should include more in-depth biological experiments to investigate these mechanisms.

## Conclusions

This study identifies three hub ferroptosis/cuproptosis-related genes and develops a prognostic signature based on these genes. Additionally, it proposes a nomogram-based model to predict OS probability for MIBC patients. The established prognostic model, along with analysis of mutational landscape, provides a valuable tool for clinical decision-making. Furthermore, the exploration of immune cell infiltration and immune checkpoint expression reveals the ferroptosis/cuproptosis-related signature’s value in predicting responses to immunotherapy and drug therapy. This represents a significant step forward in the personalized treatment of MIBC, offering hope to patients facing this aggressive disease.

### Supplementary Information

Supplementary Material 1.

Supplementary Material 2.

Supplementary Material 3.

Supplementary Material 4.

Supplementary Material 5.

Supplementary Material 6.

Supplementary Material 7.

Supplementary Material 8.

Supplementary Material 9.

Supplementary Material 10.

Supplementary Material 11.

Supplementary Material 12.

## Data Availability

No datasets were generated or analysed during the current study.

## Abbreviations

APM: Antigen processing and presenting machinery
ATCC: American type culture collection
AUC: Area under the curve
CCK-8: Cell counting kit-8
CCLE: Cancer Cell Line Encyclopedia
CI: Confidence interval
CIBERSORT: Cell-type Identification By Estimating Relative Subsets Of RNA Transcripts
CNV: Copy number variation
CRGs: Cuproptosis-related genes
CV: Cross-validation
DC: Dendritic cell
DCA: Decision curve analysis
DEA: Differential expression analysis
DECRGs: Differentially expressed cuproptosis-related genes
DEGs: Differentially expressed genes
DMEM: Dulbecco’s modified eagle medium
ESTIMATE: Estimation of STromal and Immune cells in MAlignant Tumours using Expression data
FBS: Fetal bovine serum
FC: Fold change
FRGs: Ferroptosis-related genes
GDC: Genomic Data Commons
GDSC: Genomics of Drug Sensitivity in Cancer
GEO: Gene Expression Omnibus
GISTIC: Genomic Identification of Significant Targets in Cancer
GO: Gene Ontology
GSEA: Gene set enrichment analysis
HR: Hazard ratio
IBS: Integrated Brier score
ICI: Immune checkpoint inhibitor
IIS: Immune infiltration score
IQR: Interquartile range
KEGG: Kyoto Encyclopedia of Genes and Genomes
LASSO: Least absolute shrinkage and selection operator
LMM: Linear mixed-effects model
MAF: Mutation annotation file
MC: Mast cells
MEM: Minimum essential medium
MIBC: Muscle-invasive bladder cancer
ML: Machine learning
MUFAs: Monounsaturated fatty acids
NAT: Normal adjacent tumour
NSCLC: Non-small cell lung cancer
ORA: Overrepresentation analysis
OS: Overall survival
PCD: Programmed cell death
PD-L1: Programmed death-ligand 1
PH: Proportional hazards
PPI: Protein–protein interaction
qRT-PCR: Quantitative reverse transcription polymerase chain reaction
REVIGO: Reduce and VIsualize Gene Ontology
RNA-seq: RNA sequencing
ROS: Reactive oxygen species
RPMI: Roswell Park Memorial Institute medium
RT-qPCR: Real-time quantitative polymerase chain reaction
SFA: Saturated fatty acid
si-NC: Negative control small interfering RNA
siRNA: Small interfering RNA
STRING: Search Tool for the Retrieval of Interacting Genes/Proteins
SVM-RFE: Support vector machine-recursive feature elimination
TCA: Tricarboxylic acid cycle
TCGA: The Cancer Genome Atlas
TIDE: Tumour Immune Dysfunction and Exclusion
TIGS: Tumour immunogenicity score
TMB: Tumour mutation burden
TPM: Transcripts per million
UC: Urothelial carcinoma; y/o: Years old
