# Supplementary material for: Development of prognostic model incorporating a ferroptosis/cuproptosis-related signature and mutational landscape analysis in muscle-invasive bladder cancer
Source: BMC Cancer. 2024 Aug 6;24:958. doi: 10.1186/s12885-024-12741-5 (PMC11302292; doi:10.1186/s12885-024-12741-5)

**Additional file 1:** Supplementary files

**Figure S1.** Flowchart of this study

**
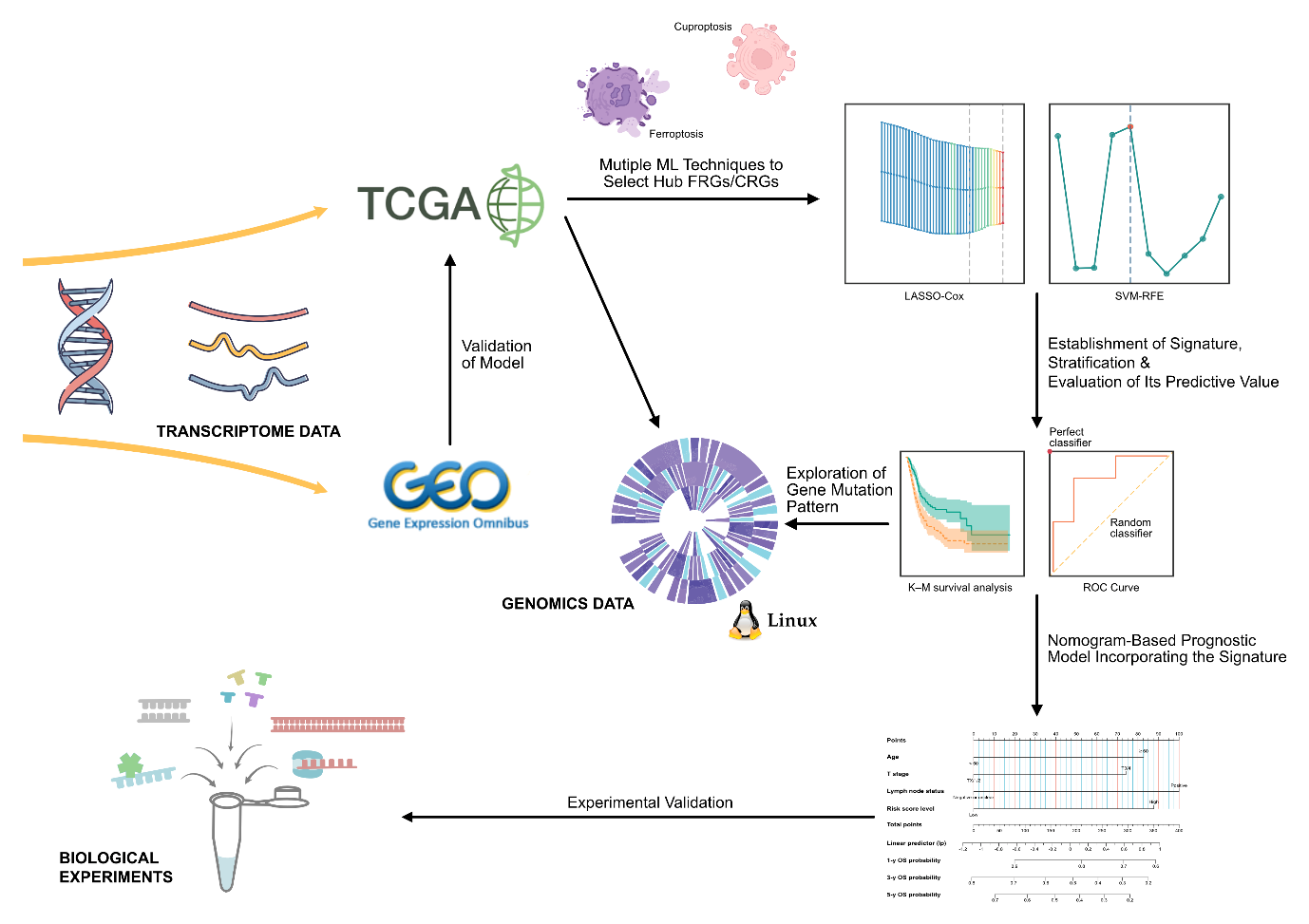
**

**Figure S2.** Single-gene gene set enrichment analysis (GSEA) for SCD in TCGA cohort. (A) Dot plot of the 20 most significantly enriched Hallmark gene sets displaying activated (red labels) and suppressed (blue labels) pathways correlated with high SCD expression, sized by gene count and colored by -log_10_(false discovery rate [FDR]). (B) Ridge plot of the 20 most significantly enriched KEGG pathways. The x-axis is the Spearman’s r from SCD co-expression analysis, and the y-axis shows the distribution of the number of genes co-expressed with SCD. Colour denoting the -log_10_(FDR).


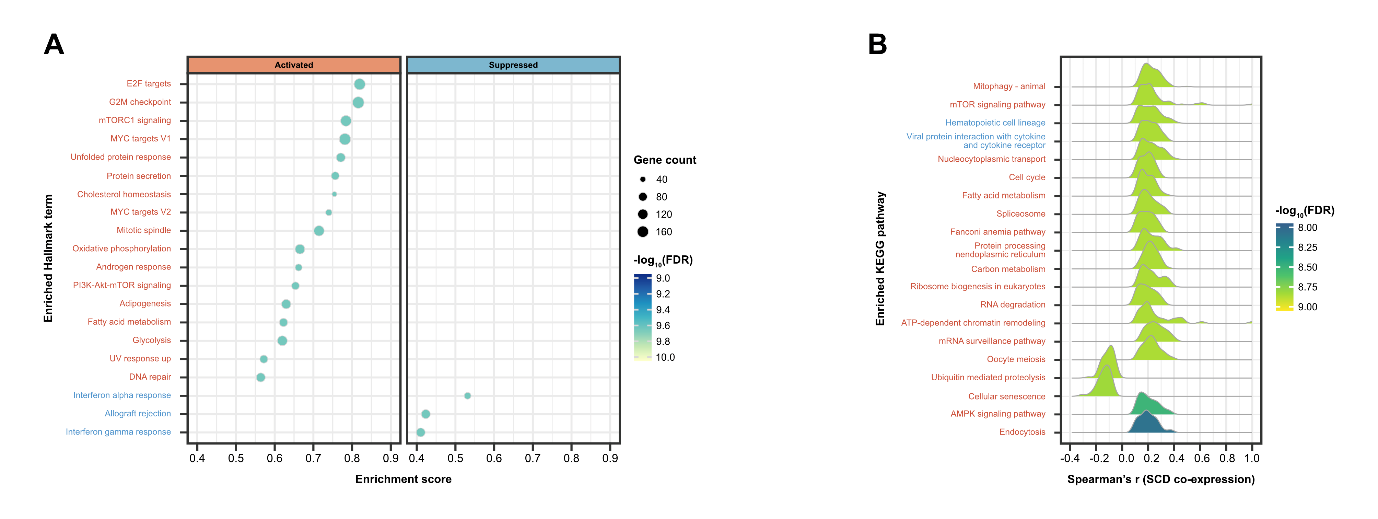

Supplement: Supplementary file 1 — Supplementary Material 1. [file 12885_2024_12741_MOESM1_ESM.docx]
