# Supplementary material for: Development of prognostic model incorporating a ferroptosis/cuproptosis-related signature and mutational landscape analysis in muscle-invasive bladder cancer
Source: BMC Cancer. 2024 Aug 6;24:958. doi: 10.1186/s12885-024-12741-5 (PMC11302292; doi:10.1186/s12885-024-12741-5)
Supplement: Supplementary file 2 — Supplementary Material 2. [file 12885_2024_12741_MOESM2_ESM.docx]

# Supplementary File 2. Supplementary Explanation of Patients and Methods

## Participants

### Data source

Raw read counts and transcript per million (TPM) normalized counts of TCGA bladder carcinoma (TCGA-BLCA) patients (platform: Illumina HiSeq 2000), somatic mutation data in mutation annotation format (MAF), gene-level copy number variation (CNV) [ Affymetrix single nucleotide polymorphism (SNP) Array 6.0], and the corresponding clinical information were sourced from the Genomic Data Commons (GDC) Data Portal (<https://portal.gdc.cancer.gov/>). The TCGA cohort, including histologically normal adjacent tumor (NAT) tissues, served as a training cohort for the development of prognostic model. The clinical traits of interest from TCGA were Vital status (Status), OS time, Age at diagnosis (Age), Gender, Race, TNM stage, T stage, Lymph node status (inferred from N stage), and Distant metastatic status (inferred from M stage). The segmental CNV data of the TCGA-BLCA cohort were simultaneously downloaded from the cBioPortal for Cancer Genomics software platform (<https://www.cbioportal.org/>) [1].

For external validation of the prognostic model established in the TCGA cohort, probe intensity matrix from BLCA patients with clinical information for GSE13507 (primary bladder cancer samples, normalized data, platform: GPL6102, Illumina human-6 v2.0 expression beadchip) [2] and GSE32894 (urothelial carcinomas [UCs], raw data, GPL6947, Illumina HumanHT-12 V3.0 expression beadchip) [3] datasets were directly obtained from GEO (<https://www.ncbi.nlm.nih.gov/geo/>). Due to the small number of patients with T3/4 in GSE32894 dataset, the prognostic model was externally validated using GSE13507 only.

A total of 581 ferroptosis-related genes (FRGs) (**Additional file 3: Table S1**) were extracted from the FerrDB V2 database (<http://www.zhounan.org/ferrdb/current/>) [4]. Additionally, the Gene Ontology (GO) term (GOBP: ferroptosis), the Wikipathway pathway (WP: ferroptosis), and the Kyoto Encyclopedia of Genes and Genomes (KEGG, https://www.kegg.jp/) [5] pathway (KEGG pathway: Ferroptosis [hsa04216]) relevant to ferroptosis (human genes only) were also included. The gene sets for the GO, Wikipathway, and Hallmark were sourced from the Molecular signatures database (MSigDB) (<https://www.gsea-msigdb.org/gsea/msigdb>) [6]. Simultaneously, a total of 85 cuproptosis-related genes (CRGs) (**Additional file 3: Table S2**) were identified through a comprehensive review of previous studies [7-13].

### Inclusion and exclusion criteria

Patients were eligible to be included in the study only if they met all of the following criteria:

1. Patients with complete survival status records and an OS time of more than 1 month (30 days).
2. Patients whose histological type was “Muscle Invasive Urothelial Carcinoma (PT2 or above)” (for the TCGA cohort only).

Patients will be excluded from the study if they met any of the following criteria:

1. Patients whose cancer tissue of origin were not located at bladder.
2. Patients with unknown TNM stage.
3. Patients with unknown grade.

The final training cohort included a total of 384 tumour samples (segmental CNV profiles: 380 tumour samples) and 17 NAT tissues from the TCGA project. GSE13507 dataset comprised 165 primary bladder cancer patients, and GSE32894 dataset consisted of 224 UC cases.

### Data processing

The duplicate ensembles/probes mapping to the same gene symbol were averaged by the “avereps” function from the “limma” package [14]. If multiple samples exist within the TCGA cohort, retain the one with a sample code that comes earliest in ascending order. log_2_ ratio-transformed TPM matrix of the TCGA samples were subjected to quantile normalization using the “normalizeBetweenArrays” function from the “limma” package [14]. For GSE32894 dataset, the “lumi” package [15] was used to carry out basic preprocessing of the raw probe intensity data, including background correcting and normalizing.

## Statistical analysis

The Kaplan–Meier (K–M) plot of survival and the Cox’s proportional hazards (PH) regression model are widely used to identify factors associated with a time-to-event response variable. The primary endpoint was overall survival (OS), defined as the time from diagnosis to death from any cause. Hazard ratio values from K–M survival curves or Cox’s PH model were used to represent the relative difference between the exposed group and the reference group. An HR value greater than 1 suggests a higher risk, while an HR value less than 1 implies a reduced risk.

Quantitative variables are presented using the mean and the standard deviation (SD) if they are normally distributed or the median and the interquartile range (IQR) if they follow a non-normal distribution. Shapiro–Wilk test was used for testing normality. Cox’s PH analyses were used to estimate the hazard ratio (HR) and their 95% confidence interval (CI) of death. The area under the curve (AUC) values of the receiver-operating characteristics (ROC) curve or concordance index (C-index) values and their 95% CI were used to display the discriminative power of the corresponding Cox’s PH model.

### Screening of genetic features

To screen hub genetic features, several regression and machine learning (ML) methods were employed to analyse the normalized TPM expression matrix through a rigorous selection process. Univariate Cox’s PH regression analyses were performed [16]. Genetic features demonstrating significant criteria (*p*-value < 0.05 and C-index > 0.5) in the univariate analysis were further subjected to advanced regression modeling. This included both a least absolute shrinkage and selection operator-penalized (LASSO-penalized) Cox’s PH regression model and a linear support vector machine (SVM) with recursive feature elimination (SVM-RFE). To optimize these models, a nested resampling cross-validation (CV) strategy was implemented, consisting of a 5-fold inner loop for hyperparameter tuning and a 5-fold outer loop for unbiased performance evaluation. Cox’s regression analyses, including calculation of the C-index, were conducted using the “survival” package [16]. ML methodologies were executed using various packages, including “mlr3” [17], “mlr3proba” [18], “mlr3verse” [19], and “mlr3extralearners” [20].

The intersection subset of features selected by two ML algorithms was further dimensionally eliminated using stepwise regression. “Cox’s.zph” function from the “survival” package [16] was used to check PH assumption of Cox’s model. Genetic features violated PH assumption, if any, were excluded. A bi-directional stepwise procedure that combined forward selection and backward elimination was utilized to refine the multivariate Cox’s PH regression analysis further, employing the “StepReg” package [21]. This procedure iteratively adjusted the model based on the Akaike information criteria (AIC) to identify the final hub genes, thereby ensuring the selection was both statistically rigorous and relevant to the biological phenomena under study.

### Internal and external validation of ferroptosis/cuproptosis-related signature

To further explore the clinical utility of the risk score, survival analyses were conducted. Independent *t*-tests compared the expression levels of hub genes across risk groups, with survival outcomes compared via Kaplan–Meier (K–M) analysis and the log-rank test, facilitated by the “coin” package [22]. The predictive accuracy of the risk score was validated both internally within the TCGA cohort and externally, assessing its performance through area under the curve (AUC) values from time-dependent receiver operating characteristic (ROC) analyses for 1, 3, and 5 years, using the “timeROC” package [23]. Signatures with an ROC AUC above 0.6 and survival analysis *p*-value below 0.05 were deemed predictive. Additionally, the risk score’s efficacy in predicting OS across subgroups defined by demographic characteristics (gender and age) was evaluated to display its potential as a robust prognostic tool.

### Validation of nomogram-based prognostic model

The performance of the nomogram-based predictive model was assessed through various metrics including the C-index for discrimination, integrated Brier score (IBS) for prediction accuracy, and calibration curves for model calibration. Additionally, decision curve analysis (DCA) [24] was used to evaluate utility of models. The discrimination and overall performance of the nomogram was evaluated on the training cohort and then validated on the validation set. IBS of the Nomogram model was computed using the “ipred” package [25] with the parameter nbagg = 1 (a single survival tree). The upper and lower 95% CI were calculated by 1000-time bootstrap resampling using the “boot” package [26]. In practice, a model with a C-index greater than 0.6 and an IBS below 0.25 was deemed useful. Meanwhile, calibration plots were generated based on 1000 bootstrap samples from both the TCGA cohort and GSE21257 dataset to compare the K–M method estimated and nomogram-predicted 1-, 3-, and 5-year OS rates using the “riskRegression” package [27]. Time-dependent C-index values involving CV with 1000 bootstrap samples of each Cox’s regression model (the Clinical model, the Risk Score Level model, and Nomogram model) were calculated utilizing the “pec” package [28]. Another novel discriminative performance measurement method, DCA, was simultaneously employed to internally validate the nomogram. Decision curves were constructed using the “stdca.R” script [29] to evaluate the 1-, 3-, and 5-year performance of each model on the training set.

**References**

1. Gao J, Mazor T, Ciftci E, Raman P, Lukasse P, Bahceci I et al. The cBioPortal for Cancer Genomics: an intuitive open-source platform for exploration, analysis and visualization of cancer genomics data. Cancer Res. 2018;78(13_Supplement):923.

2. Kim WJ, Kim EJ, Kim SK, Kim YJ, Ha YS, Jeong P et al. Predictive value of progression-related gene classifier in primary non-muscle invasive bladder cancer. Mol Cancer. 2010;9(1):3.

3. Sjödahl G, Lauss M, Lövgren K, Chebil G, Gudjonsson S, Veerla S et al. A molecular taxonomy for urothelial carcinoma. Clin Cancer Res. 2012;18(12):3377–86.

4. Zhou N, Yuan X, Du Q, Zhang Z, Shi X, Bao J et al. FerrDb V2: update of the manually curated database of ferroptosis regulators and ferroptosis-disease associations. Nucleic Acids Res. 2023;51(D1):D571–82.

5. Kanehisa M, Goto S. KEGG: Kyoto Encyclopedia of Genes and Genomes. Nucleic Acids Res. 2000;28(1):27–30.

6. Liberzon A, Subramanian A, Pinchback R, Thorvaldsdóttir H, Tamayo P, Mesirov JP. Molecular signatures database (MSigDB) 3.0. Bioinformatics. 2011;27(12):1739–40.

7. Luo B, Lin J, Ni A, Cai W, Yu X, Wang M. A novel defined cuproptosis-related gene signature for predicting the prognosis of colon adenocarcinoma. Front Oncol. 2022;12:927028.

8. Huang Y, Yin D, Wu L. Identification of cuproptosis-related subtypes and development of a prognostic signature in colorectal cancer. Sci Rep. 2022;12(1):17348.

9. Liu Y, Liu Y, Ye S, Feng H, Ma L. Development and validation of cuproptosis-related gene signature in the prognostic prediction of liver cancer. Front Oncol. 2022;12:985484.

10. Cai Z, He Ye, Yu Z, Hu J, Xiao Z, Zu X et al. Cuproptosis-related modification patterns depict the tumor microenvironment, precision immunotherapy, and prognosis of kidney renal clear cell carcinoma. Front Immunol. 2022;13:933241.

11. Tsvetkov P, Coy S, Petrova B, Dreishpoon M, Verma A, Abdusamad M et al. Copper induces cell death by targeting lipoylated TCA cycle proteins. Science. 2022;375(6586):1254–61.

12. Dong J, Wang X, Xu C, Gao M, Wang S, Zhang J et al. Inhibiting NLRP3 inflammasome activation prevents copper-induced neuropathology in a murine model of Wilson’s disease. Cell Death Dis. 2021;12(1):87.

13. Ge EJ, Bush AI, Casini A, Cobine PA, Cross JR, DeNicola GM et al. Connecting copper and cancer: from transition metal signalling to metalloplasia. Nat Rev Cancer. 2022;22(2):102–13.

14. Ritchie ME, Phipson B, Wu D, Hu Y, Law CW, Shi W et al. *limma* powers differential expression analyses for RNA-sequencing and microarray studies. Nucleic Acids Res. 2015;43(7):e47.

15. Du P, Kibbe WA, Lin SM. *lumi*: a pipeline for processing Illumina microarray. Bioinformatics. 2008;24(13):1547–8.

16. Therneau TM, Grambsch PM. The Cox model. In: Modeling survival data: extending the Cox model. New York, NY: Springer New York; 2000. pp. 39–77.

17. Lang M, Binder M, Richter J, Schratz P, Pfisterer F, Coors S et al. mlr3: a modern object-oriented machine learning framework in R. J Open Source Softw. 2019;4(44):1903.

18. Sonabend R, Király FJ, Bender A, Bischl B, Lang M. mlr3proba: an R package for machine learning in survival analysis. Bioinformatics. 2021;37(17):2789–91.

19. Lang M, Schratz P. mlr3verse: easily install and load the ‘mlr3’ package family; 2023. Available from: <https://github.com/mlr-org/mlr3verse>. Accessed July 15 2023.

20. Sonabend R, Schratz P, Fischer S. mlr3extralearners; 2023. Available from: <https://github.com/mlr-org/mlr3extralearners>. Accessed July 15 2023.

21. Li J, Lu X, Cheng K, Liu W. Package ‘StepReg’. Boston, MA: CRAN; 2022. Available from: <https://cran.r-project.org/web/packages/StepReg/StepReg.pdf>. Accessed June 15, 2023.

22. Hothorn T, Hornik K, Van De Wiel MA, Zeileis A. Implementing a class of permutation tests: the coin package. J Stat Softw. 2008;28(8):1–23.

23. Blanche P, Dartigues J-F, Jacqmin-Gadda H. Estimating and comparing time-dependent areas under receiver operating characteristic curves for censored event times with competing risks. Stat Med. 2013;32(30):5381–97.

24. Vickers AJ, Elkin EB. Decision curve analysis: a novel method for evaluating prediction models. Med Decis Making. 2006;26(6):565–74.

25. Peters A, Hothorn T, Lausen B. ipred: Improved predictors. R News. 2002;2(2):33–6.

26. Canty AJ. Resampling methods in R: the boot package. Newsl R Proj. 2002;2(3):2–7.

27. Lazic SE. Medical risk prediction models: with ties to machine learning. J R Stat Soc Ser A Stat Soc. 2022;185(1):425.

28. Mogensen UB, Ishwaran H, Gerds TA. Evaluating random forests for survival analysis using prediction error curves. J Stat Softw. 2012;50(11):1–23.

29. Vickers AJ, Cronin AM, Elkin EB, Gonen M. Extensions to decision curve analysis, a novel method for evaluating diagnostic tests, prediction models and molecular markers. BMC Med Inform Decis Mak. 2008;8(1):53.
